# mRNA Profiling and Transcriptomics Analysis of Chickens Received Newcastle Disease Virus Genotype II and Genotype VII Vaccines

**DOI:** 10.3390/pathogens13080638

**Published:** 2024-07-30

**Authors:** Putri Pandarangga, Phuong Thi Kim Doan, Rick Tearle, Wai Yee Low, Yan Ren, Hanh Thi Hong Nguyen, Niluh Indi Dharmayanti, Farhid Hemmatzadeh

**Affiliations:** 1 Departemen Klinik, Reproduksi, dan Patologi, Fakultas Kedokteran dan Kedokteran Hewan, Universitas Nusa Cendana, Kupang 85001, Indonesia; putri.pandarangga@gmail.com; 2 School of Animal and Veterinary Sciences, University of Adelaide, Adelaide 5371, Australia; phuong.doan@adelaide.edu.au (P.T.K.D.); hanh.t.nguyen@adelaide.edu.au (H.T.H.N.); 3 Department of Veterinary Medicine, Tay Nguyen University, Buon Ma Thuot 630000, Vietnam; 4 Davies Research Centre, School of Animal and Veterinary Sciences, University of Adelaide, Adelaide 5371, Australia; rick.tearle@adelaide.edu.au (R.T.); wai.low@adelaide.edu.au (W.Y.L.); kelly.ren@adelaide.edu.au (Y.R.); 5 National Research and Innovation Agency, Jakarta 10340, Indonesia; nlpdharmayanti@gmail.com

**Keywords:** transcriptomic, vaccine, NDV genotype II, NDV genotype VII, synaptogenesis pathway

## Abstract

Newcastle Disease Virus (NDV) genotype VII (GVII) is becoming the predominant strain of NDV in the poultry industry. It causes high mortality even in vaccinated chickens with a common NDV genotype II vaccine (GII-vacc). To overcome this, the killed GVII vaccine has been used to prevent NDV outbreaks. However, the debate about vaccine differences remains ongoing. Hence, this study investigated the difference in chickens’ responses to the two vaccines at the molecular level. The spleen transcriptomes from vaccinated chickens reveal that GVII-vacc affected the immune response by downregulating neuroinflammation. It also enhanced a synaptogenesis pathway that operates typically in the nervous system, suggesting a mechanism for the neurotrophic effect of this strain. We speculated that the down-regulated immune system regulation correlated with protecting the nervous system from excess leukocytes and cytokine activity. In contrast, GII-vacc inhibited apoptosis by downregulating PERK/ATF4/CHOP as part of the unfolded protein response pathway but did not affect the expression of the same synaptogenesis pathway. Thus, the application of GVII-vacc needs to be considered in countries where GVII is the leading cause of NDV outbreaks. The predicted molecular signatures may also be used in developing new vaccines that trigger specific genes in the immune system in combating NDV outbreaks.

## 1. Introduction

Newcastle Disease (ND) is caused by the velogenic Newcastle Disease Virus (vNDV), a major transboundary animal disease of chickens that engenders large economic losses annually [1]. This virus belongs to the genus *Orthoavulavirus* in the family *Paramyxoviridae* [2] and has caused multiple panzootic events since 1926 [3]. In the latest phylogenetic classification of avian paramyxoviruses, NDV class II contains twenty-one genotypes that can be velogenic, mesogenic, or lentogenic. While the velogenic neurotropic or viscerotropic strains have higher virulence than lentogenic ones [4]. The most pathogenic NDV strains, which have caused outbreaks worldwide, including in Africa [5,6,7,8], Asia [9,10], Eastern Europe [7,11,12], and South America [13] belong to genotype VII (GVII) [14,15,16,17]. However, the commercial vaccines in use since the 1950s are derived from genotypes I (GI) and II (GII) [18]. GII and GVII are substantially different, with 21.6% of nucleotides discordant, where 10% is the threshold to establish a new genotype [4,19].

The antigenic difference between the NDV vaccine and NDV circulating in the field is likely one of the main reasons that ND outbreaks continue [20,21]. In Southeast Asia, the mortality of GII-vaccinated chickens reaches 70–80%, which has been proposed due to antigenic differences that affect the ability of the commercial vaccine to protect chickens [6,22,23]. To overcome the NDV outbreaks caused by NDV an effective vaccine needs to be developed. However, antigenic differences as a reason for vaccine failure are still a topic of concern amongst NDV researchers [5]. Multiple experimental studies on NDV-GVII-vacc showed homologous vaccines can protect chickens better than GII vaccines. The GVII vaccines provide longer immunity and casing less virus shed in challenge experiments [20,24,25,26]. Despite this, Cornax et al. (2012) hypothesized that the GII-vacc known as the LaSota vaccine can still protect chickens from GVII as long as it is applied correctly and no maternal antibodies are present [27]. Moreover, the commercial vaccine can still reduce viral shedding after the challenge of virulent NDV [28]. However, these studies only considered serology, clinical signs, and viral load count, limiting the consideration of a vaccine’s applicability.

Multiple research groups have applied transcriptomics analysis to understand the molecular basis of NDV pathogenesis, however, have not identified molecular signatures in vaccinated chickens using the NDV vaccine. Furthermore, to the best of our knowledge, this comparison is a novel approach to understanding the NDV-GVII vaccines and hasn’t been published elsewhere [29]. In virulent NDV infection, the activation genes are related to autophagy-mediated cell death, lymphotropic, and synaptogenesis signaling pathway [30]. On the other hand, non-virulent NDV infection, namely LaSota, tends to activate interferon stimulating genes (ISGs), control the pathways such as actin cytoskeleton regulation and mitogen-activated protein kinase (MAPK) signaling correlated to changing extracellular signal into a cellular response [31].

The mechanisms whereby non-virulent and virulent NDV vaccines stimulate the chicken immune system remain obscure. Furthermore, molecular signatures, consisting of pathways and genes that protect chickens, have not been thoroughly investigated for GII-vacc and GVII-vacc. Hence, this study aimed to identify the significant pathways and genes in chickens, especially in the spleen, after the second injection of the GII-vacc or GVII-vacc to identify molecular signatures for each. Histopathological studies on experimentally infected chickens with the NDV-GVII have shown that the spleen and Bursa of Fabricius are the main lymphoreticular organs that are heavily invaded by the GVII viruses. These findings have been reported in a few different observational and retrospective studies by Ni et al., (2023), Sultan et al. (2020), and Liu et al., (2023) [16,20,30,32,33]. To clarify this behavior and change of the GVII viruses, we targeted the spleen as the main lymphoreticular organ to compare the effects of GII versus GVII NDVs in the spleen tissue of vaccinated chickens.

Moreover, based on ingenuity pathway analysis (IPA) prediction, we aimed to highlight the difference between host response, including immune regulation, to the commercial vaccine and GVII-vacc as a homologous vaccine at the molecular level. This knowledge may lead to new insights into developing potent vaccines by targeting the specific genes that stimulate transcription factors related to chickens’ immune responses.

## 2. Materials and Methods

### 2.1. Viruses and Vaccines

Ethical clearance of the animal experiment with number AH/2015/003 was approved by the Indonesian Research Center for Veterinary Science’s (BBALITVET) animal ethics committee. The animals were monitored by a veterinarian and a veterinary pathologist by following the guidelines of the National Health and Medical Research Council of Australia. For this study’s challenge experiments and vaccine preparation, two viral stocks were used. LaSota-GII, sourced from the NDV archive at the Indonesian Research Center for Veterinary Science. The LaSota-GII is a common strain used for vaccines in Indonesia and provided by major vaccine producers worldwide [23]. The NDV-GVII isolate named chicken/Indonesia/Mega/001WJ/2013 was isolated from an outbreak in 2011 in West Java, Indonesia, and characterized by Doan et al. (2020) as a virulent NDV strain [34,35]. Each isolate was propagated in 9-day-old SPF embryonated chicken eggs [36]. Haemagglutination (HA) test and qPCR were used to confirm the presence of NDV in the allantoic fluid. Reed and Muench’s method was used to calculate 50% embryo infection dose (EID_50_) as the required virus titer for vaccine production [37,38]. Sterile Phosphate-Buffered Saline (PBS) pH 7.2 was used to adjust the titer to 10^7^ EID_50_ in each dose of the vaccines [39]. To inactivate the diluted viruses, 0.12% formalin was utilized for 12 h at 4 °C [36,40,41]. The inactivation of the virus was confirmed by inoculating diluted viruses into 10 SPF chicken eggs (100 μL/egg). The eggs were kept for 5 days in a 37 °C chicken egg incubator and checked daily for any embryo death. On the day 6th, the allantoic fluid of the eggs was tested in HA tests. No dead embryos and no positive HA were signs that the virus was properly inactivated. Each vaccine dose contained 0.5 mL incomplete Freund’s adjuvant and 0.5 mL inactivated virus.

### 2.2. Animal Experiments

A total of thirty, three-week-old specific-pathogen-free (SPF) chickens, defined as day 0, were divided into three groups of 10 and placed in separate isolators. All chickens were raised with ad libitum access to feed and water. The first group was used as a control group that was injected with phosphate-buffered saline (PBS) (*n* = 10). The second group was vaccinated with the LaSota GII vaccine, hereafter referred to as GII-vacc (*n* = 10). The last group was vaccinated using the GVII-vacc (*n* = 10). The route of vaccination was intra-muscular at the breast muscle. Treatment groups were immunized with vaccines on day 14 and day 28 [26,30]. On day 42, all the groups were euthanized using CO_2_. After a systematic necropsy, from each bird, ~100 mg of spleen was taken and immediately stored in 1 mL RNAlater^TM^ in a 2-mL tube (ThermoFisher Scientific, Waltham, MA, USA) at −80 °C. Viral loading and serology data were calculated for each serum and tissue sample collected in this study [26]. The serology results have been published to support the comparison of GII-vacc and GVII-vacc at the molecular level [26,30].

### 2.3. RNA Preparation and Sequencing

Total RNA was extracted and purified from spleen samples using *mir*Vana™ miRNA Isolation Kit based on the manufacturer’s instructions (ThermoFisher Scientific, Vilnius, Lithuania). To eliminate genomic DNA (gDNA), the total RNA was incubated in a gDNA buffer at 42 °C for 2 min. The quantity of RNA was measured using a NanoDrop 1000 Spectrophotometer v 3.8 (ThermoFisher Scientific). Moreover, the RNA Integrity Number (RIN) was validated by using Agilent 2200 TapeStation (Agilent Technologies, Santa Clara, CA, USA). To avoid any biases in RNAseq results the RNA samples, a high quality of RNA with a similar RIN were used for further analysis. Library preparation and sequencing were performed at the Australian Genome Research Facility (AGRF). RNA quality (RIN score) of 8 samples was measured using a LabChip GX Touch (Lab Chip Technologies, Edmonton, AB, Canada) nucleic acid analyzer (PerkinElmer, Calgary, AB, Canada). The range of RIN scores was 5.6–10.0. Based on the RIN score and the Nanodrop reads, 6 samples with the higher RIN scores were selected for the next step. Complementary DNA (cDNA) libraries were prepared using a KAPA-stranded RNA-seq kit (Roche, Atlanta, GA, USA). Finally, the libraries were sequenced using an Illumina NovaSeq (Illumina, San Diego, CA, USA) with a NovaSeq 6000 S4 Reagent Kit. After a comparison of all quality control measures, only three samples from each experimental group with the highest RIN, similar RNA concentration, and similar read distribution were selected for transcriptomics analysis.

### 2.4. Transcriptome and Pathway Analysis of Vaccinated Chickens Differential Expression Genes (DEGs)

The quality of raw data was assessed using FASTQC v 0.11.4 and trimmed with TrimGalore v 0.4.2 to a minimum length of 100 bp, and a minimum sequencing quality of Phred score 10. Sequencing adapters were removed with AdapterRemoval v 2.2.1 [42]. The reads were aligned to the chicken reference genome (GRCg6a) using Hisat2 v 2.2.1, sorted, merged, and indexed using SAMtools v 1.8 [43]. FeatureCounts was used to summarize counts of reads mapped to genes using Ensembl Annotation v 97 [44]. Voom-limma was used to compare samples grouped by vaccination status [45,46]. Read counts were converted to counts per million (CPM), and only genes with more than 1 CPM in at least three samples were kept. Counts were normalized by log-transforming the CPM after correcting for differences in library size. Counts were further normalized using M values’ trimmed mean (TMM) [47]. Samples and individual observational levels of each expressed gene were weighted using Voom to adjust for heterogeneity in their expression level [46]. Differentially expressed genes (DEGs) between groups were identified using a False Discovery Rate (FDR) of less than 0.05. The DEGs from the three group comparisons were analyzed using Ingenuity Pathway Analysis (IPA) software version 2.2.1 from QIAGEN (Hilden, Germany) to find significantly differentially expressed genes and pathways that correlate with the chicken immune response.

### 2.5. Validation of RNA Sequencing Using Quantitative PCR (qPCR)

Seven genes from the DEGs list with an absolute Log Fold Change (LFC) greater than 1.6 were chosen for validation by quantitative PCR (qPCR). Primer-BLAST was used to find specific primers for each gene. The primers were designed to span exon-exon junctions to avoid amplification of genomic DNA. The range of PCR products was ~70–250 base pairs. The optimal range of primer melting temperature was between 58–64 °C. RNA was converted to cDNA using a SuperScript IV Reverse Transcriptase kit (ThermoFisher Scientific), following the protocol recommended by the manufacturer. Quantitative PCR was performed using a QuantiNova SYBR^®^ Green RT-PCR kit (Qiagen, Hilden, Germany). The ΔΔCt method was used to quantify the expression using log fold change. GAPDH and ACTB were used as reference genes for normalization. The relationship between the expression of the genes from the transcriptome and qPCR was estimated using Pearson correlation.

## 3. Results

### 3.1. RNA-Sequencing Reads

**Figure 1:**
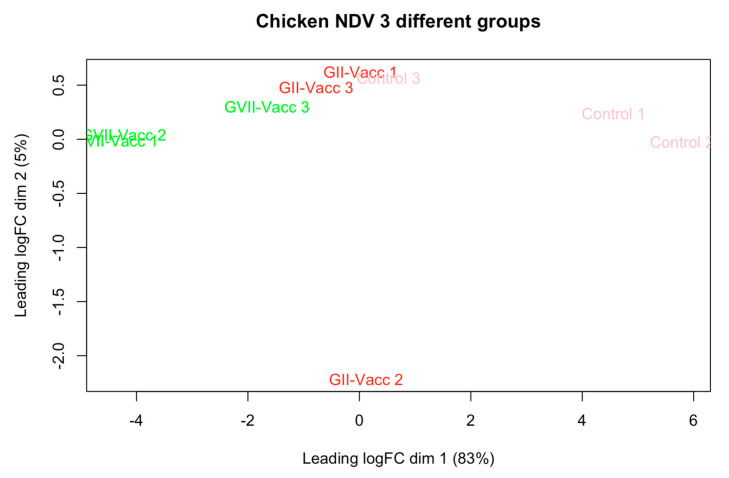
PCA plot for the three experimental groups as per Table 1. The groups are included including vaccinated chickens with NDV-GVII, NDV-GII, and unvaccinated chickens as control groups.

Due to RNA quality, three samples from each with the best quality and score of RNA integrity number (RIN) were selected for RNA sequencing and transcriptomics analysis. The intergroup data homogeneity was analyzed to ensure the quality of the data for further analysis. Figure 1 shows the PCA plot for the quality control of the data. While there was intragroup variation, the clustering of the three experimental groups could be seen.

Counts of RNA-seq reads from chicken splenic RNA across the three groups are summarized in Table 1. Up to 16% of raw reads were removed during filtering and data cleaning to reach an acceptable proportion [48]. Thus, up to 89% of clean reads were mapped to the chicken genome (GRCg6a). A total of 15,355 genes were expressed across all the groups.

### 3.2. Gene Expression Induced by GII-Vacc and GVII-Vacc

**Figure 2:**
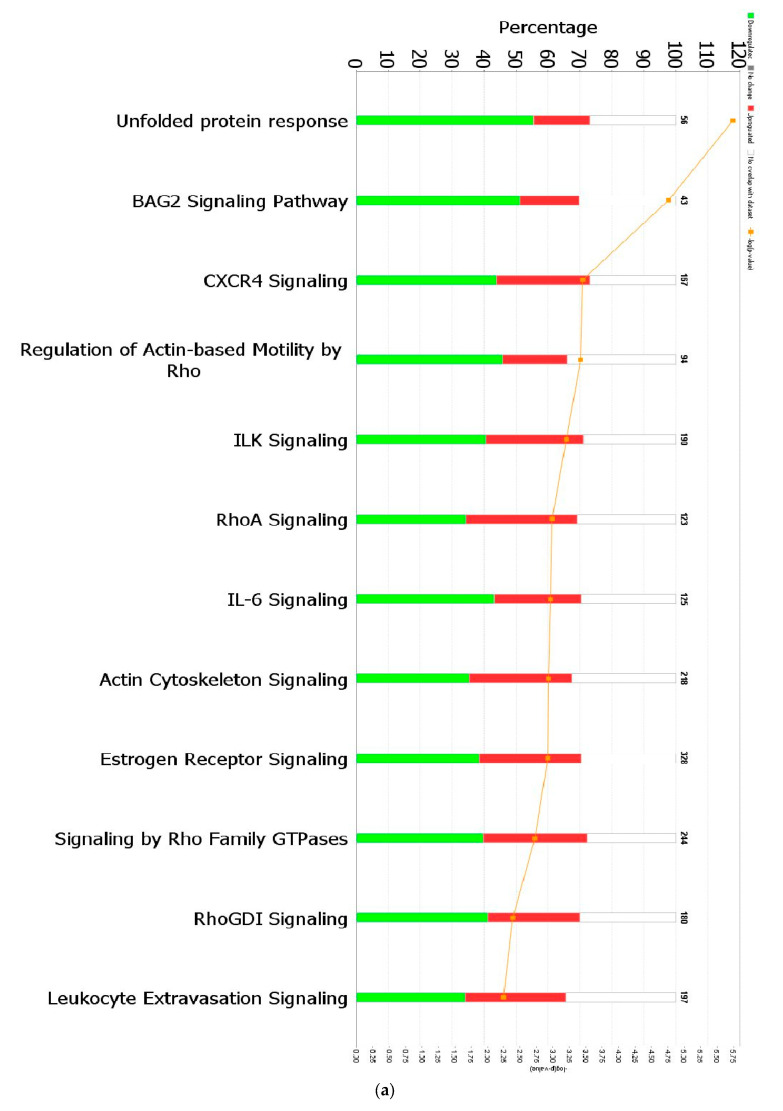

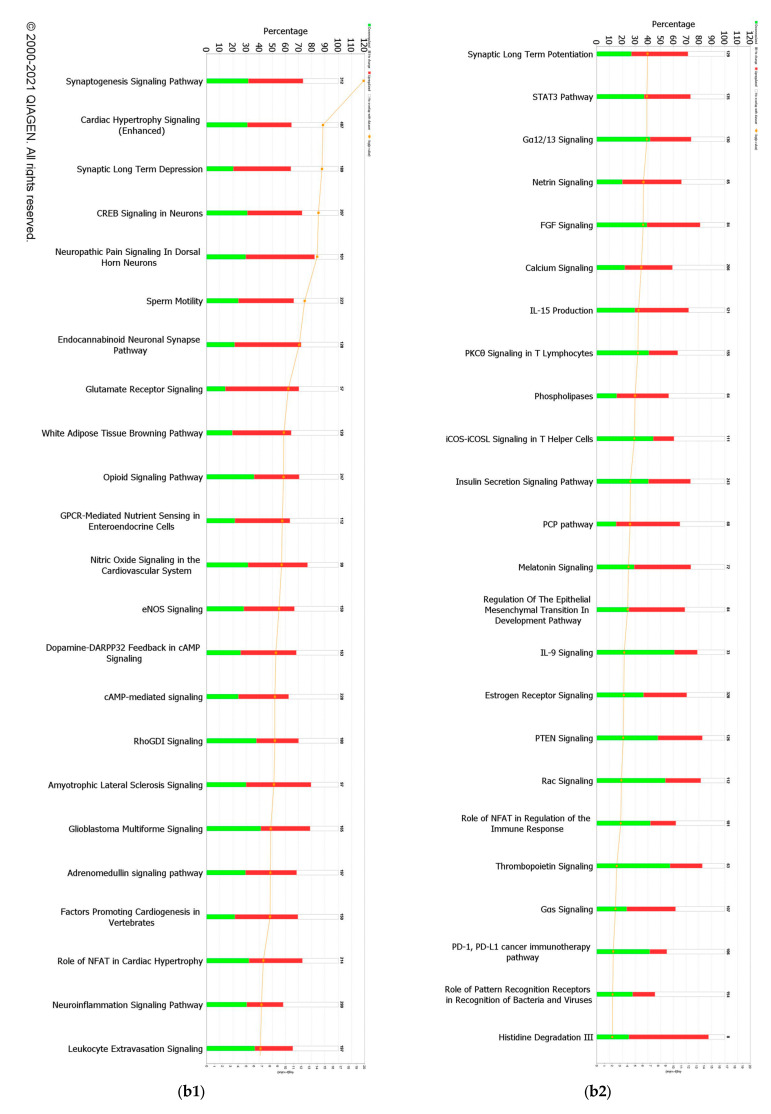

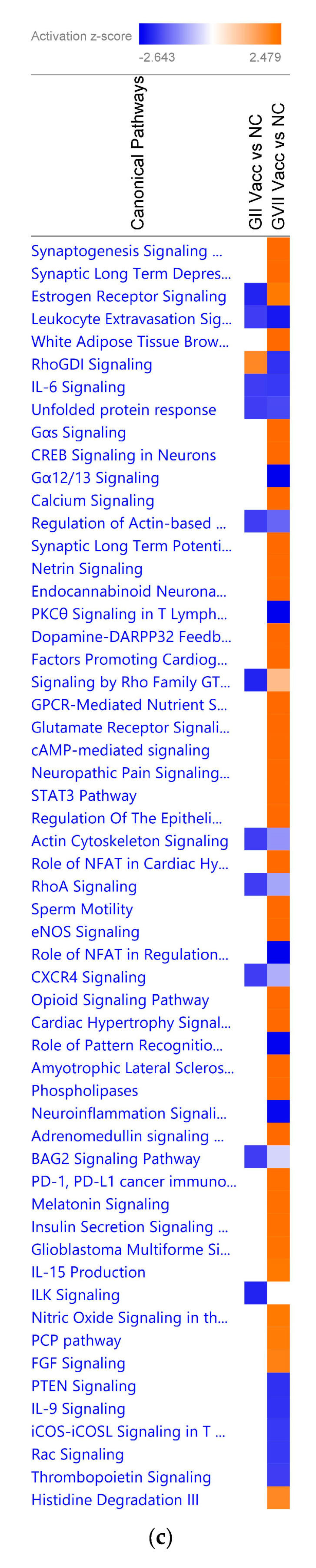
Predicted significant pathways in chicken response to NDV GII and GVII vaccines: (**a**) Twelve significant pathways from GII-vacc DEGs; (**b1**,**b2**) Forty-seven pathways from GVII-vacc DEGs. DEGs were selected with a-log (*p*-value) > 2 and an absolute z-score was >2 as a cutoff. The number on the top of each bar represents the total number of genes contributing to the pathways. Each bar has three colors: green, red, and white, represented as down-regulated, up-regulated, and no overlap genes with the basic knowledge in IPA. The –log (*p*-value) of each pathway is indicated by the orange line; (**c**) Heatmap. Comparing GII-vacc and GVII-vacc DEGs shows orange boxes as activated pathways, blue boxes as inhibited pathways, and white boxes as non-significant pathways. These pathways are filtered with a –log (*p*-value) > 2.5 and z-score > 2. The contrast in the heatmap was visualized with a z-score feature (High-quality figures are available in Supplementary Files).

When a False Discovery Rate (FDR) of ≤0.05 and log fold change of ±1 were set as thresholds, only 89 genes from GII-vacc vs. control and 2751 genes from GVII-vacc vs. control were reported as DEGs. Using 2 as an absolute z-score, 12 significantly differentially expressed pathways were identified for GII-vacc vs. control (Figure 2a) and 43 for GVIIvacc vs. control (Figure 2(b1,b2)). Each pathway contained up-and down-regulated genes (Supplementary Table S1).

### 3.3. Ingenuity Pathway Analysis of Spleen of Chickens Vaccinated with GII-Vacc

Chickens that were vaccinated with GII-vacc showed 12 differentially regulated pathways (Figure 2a). These pathways fall into two main groups, immune response regulation and essential cellular functions. The immune response regulation group contained cytokine signaling genes (CXCR4 signaling and IL-6) and cellular immune response genes (leukocyte extravasation genes). All the pathways in this group were down-regulated. The basic cellular function group contained: (1) cellular stress and injury genes, (2) cellular growth, proliferation, and development genes, and (3) intracellular and second messenger genes. Similar to the immune response regulation group, all the pathways in these groups were down-regulated except for the RhoGDI signaling pathway (Figure 2c). In addition, individual genes that have a role in controlling immune response were down-regulated such as CEBPB, CXCR4, and THY1 (Supplementary Table S1).

**Figure 3:**
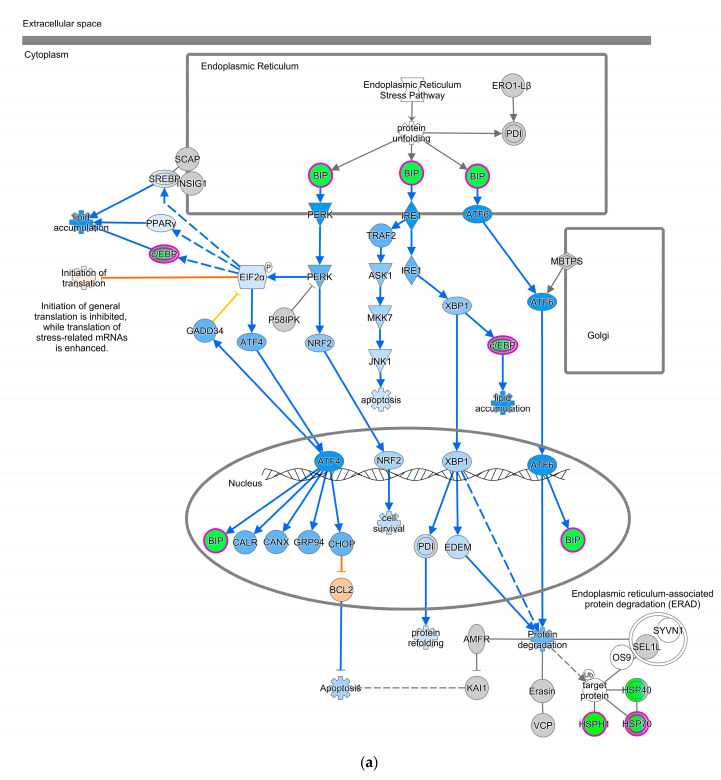

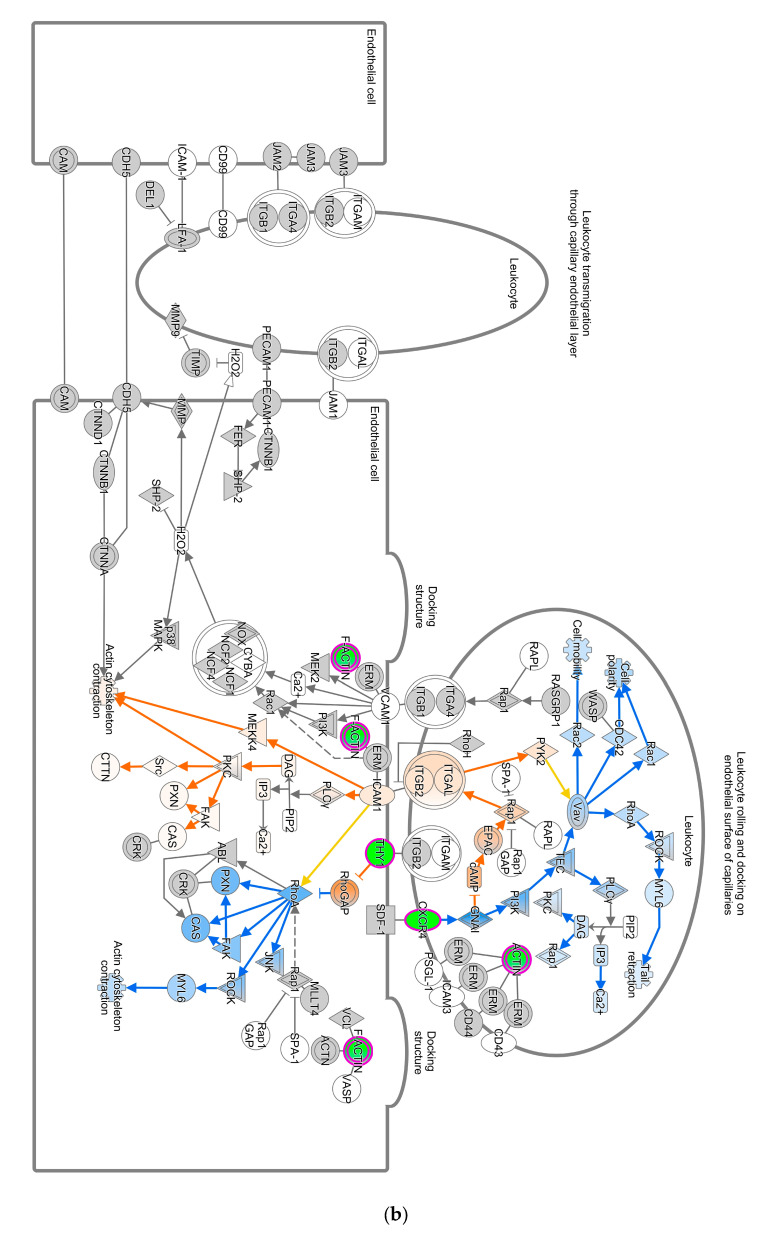
Pathways altered in chickens vaccinated with GII. The pathways are overlaid with the Molecule Activity Predictor (MAP) feature to recognize the unknown molecules. Green and red shapes represent down- and up-regulation. The orange and blue shapes represent predicted molecule activation and inhibition, respectively. (**a**) Unfolded Protein Response (UPR) pathway. (**b**) Leukocyte extravasation signaling.

The Unfolded Protein Response (UPR) pathway (Figure 3a) is part of the cellular stress and injury group. This pathway regulated responders to Endoplasmic Reticulum (ER) stress, including stress transducers such as PERK, IRE1, and ATF6. All these transducers were down-regulated (blue color in Figure 3a). PERK is a part of EIF2α and was down-regulated, suppressing the protein that causes apoptosis. XBP-1, a gene downstream of IRE1, was down-regulated. In addition, actin cytoskeleton signaling, regulation of actin-based motility by Rho, and ILK signaling involved in cellular growth, proliferation, and development were down-regulated (not depicted). As part of the intracellular and second messenger group, GTPase signaling family genes such as Rac, Rho, and cdc42 played a dominant role in cellular development pathways in chickens vaccinated with GII-vacc. Moreover, the activities of the family GTPase as the second messenger were down-regulated in the leukocyte extravasation pathway (Figure 3b).

### 3.4. Ingenuity Pathway Analysis of Chickens Vaccinated with GVII-Vacc

A total of forty-seven pathways were differentially regulated in the GVII-vacc vs. control (Figure 2b). These pathways were grouped into (1) immune system regulation, consisting of cellular immune response (neuroinflammation signaling pathway, leukocyte extravasation, and regulation of T lymphocytes), cytokine signaling (IL-6 and IL-9 signaling), and humoral immune response (NFAT regulating the immune response); (2) essential cellular functions comprised UPR pathway as stress and injury cell response; and (3) nervous system signals containing synaptogenesis signaling, long-term depression (LTD), and long-term potentiation (LTP) signaling.

**Figure 4:**
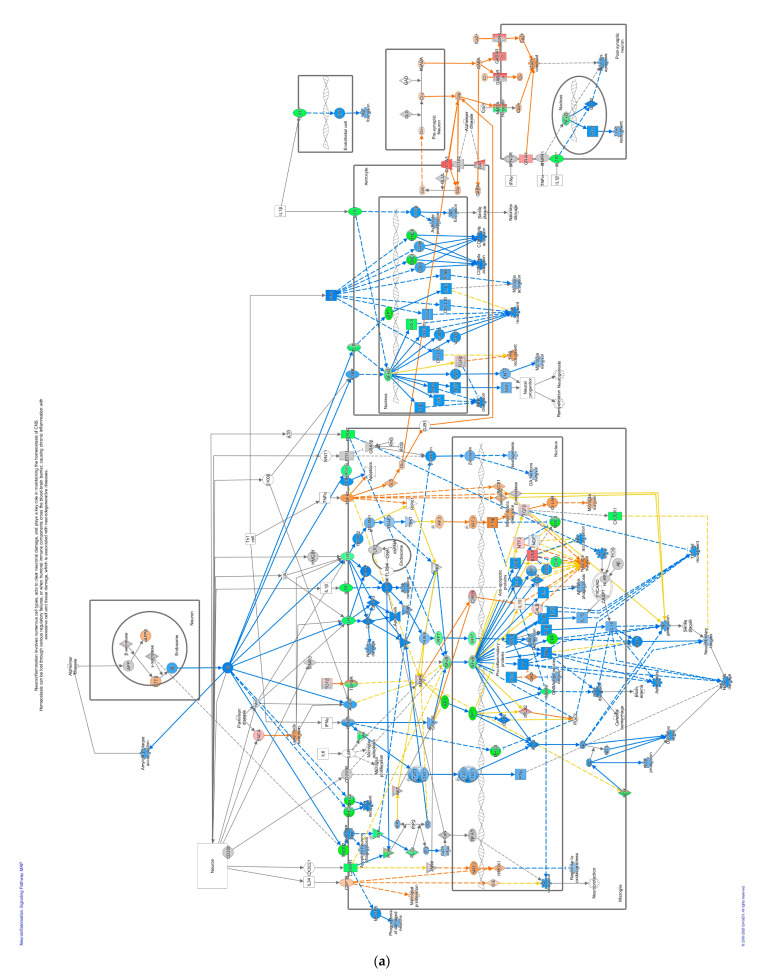

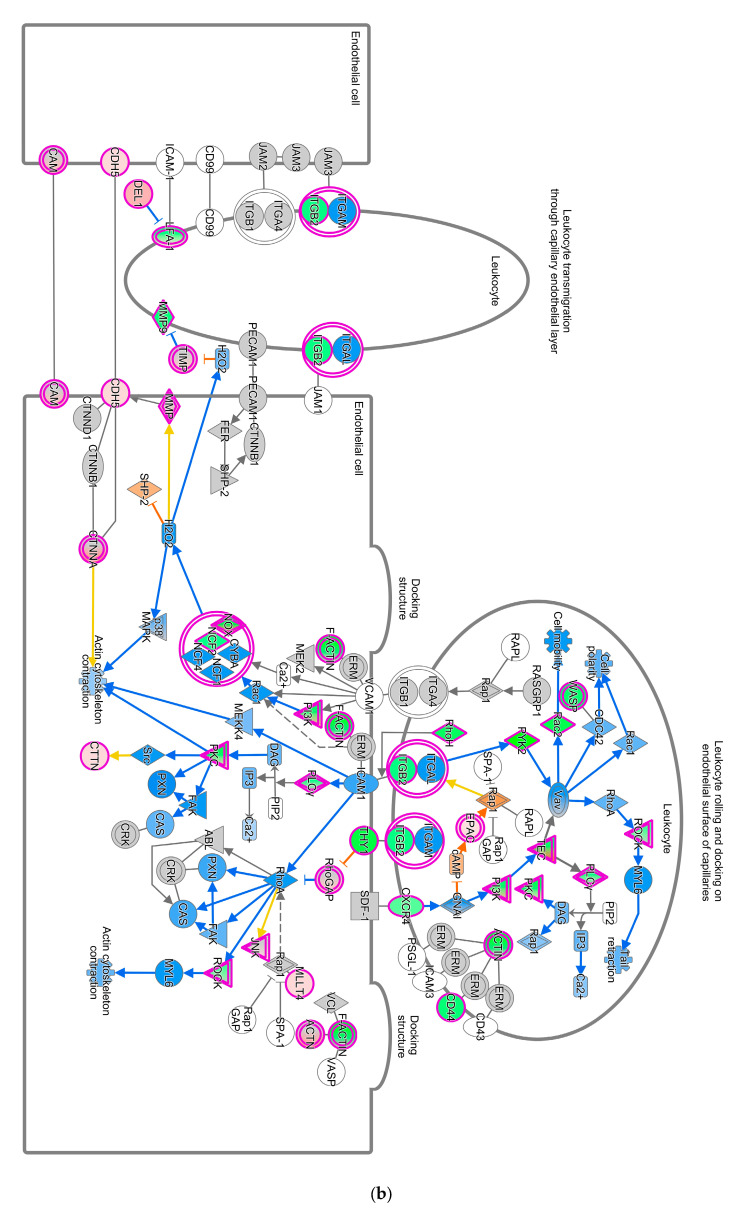

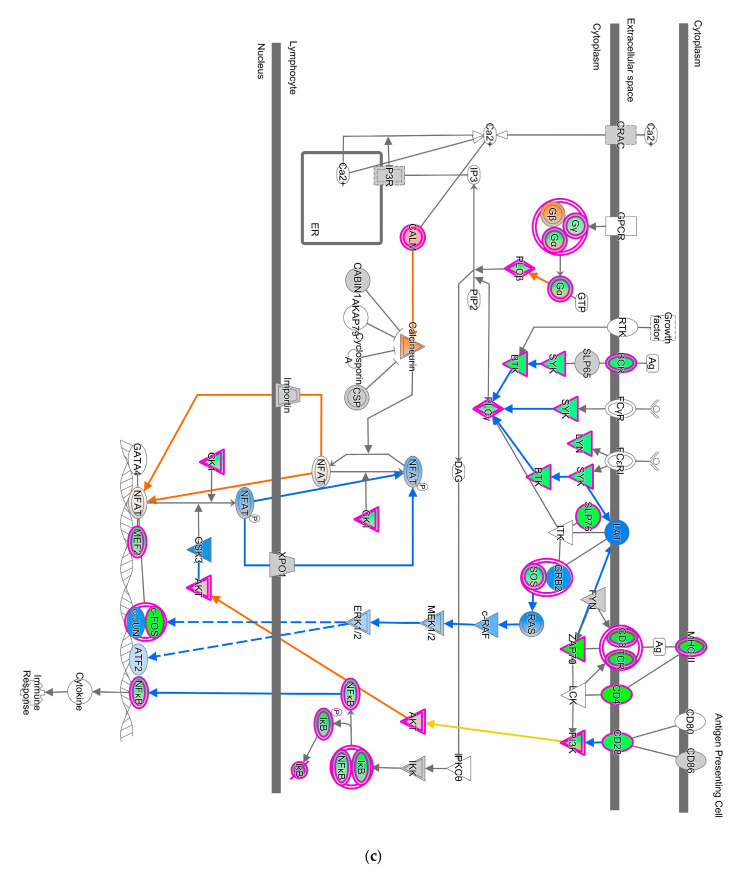

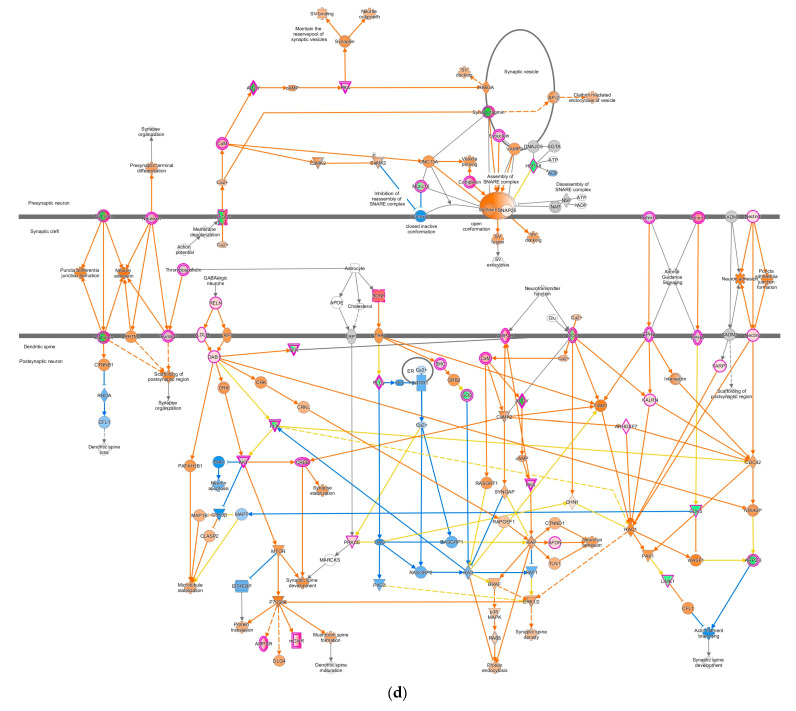
Pathway altered in chickens vaccinated with GVII. The pathways were overlaid with the IPA Molecule Activity Predictor (MAP) feature to recognize the unknown molecules. Green and red shapes represent down- and up-regulation. The orange and blue shapes represent the predicted activation and inhibition of molecules. (**a**) The neuroinflammation signaling pathway; (**b**) the leukocyte extravasation; (**c**) the role of NFAT in regulating the immune response; and (**d**) the synaptogenesis signaling pathway.

Pathways that correlated with immune system regulation were down-regulated, including the neuroinflammation signaling pathway (Figure 4a), leukocyte extravasation (Figure 4b), and NFAT-regulating immune response (Figure 4c). NF-ƙB (marked with green circles) in microglial cells was a part of neuroinflammation signaling (Figure 4a). The down-regulated NF-ƙB is capable of inhibiting the activities of downstream proteins such as IAP, BCL-2, pro-inflammatory, and anti-apoptotic proteins shown with blue squares. However, the downregulated NF-ƙB can also activate NTF3 in microglia cells, as shown in a pink square (Figure 4a). In the neuroinflammation pathway, IFN γ in splenocytes was downregulated and prevented the activity of downstream proteins such as T cell, CD4+, CD8+ recruitments, and microglia activation that showed with blue shapes (Figure 4a).

In contrast, the pathways involved in nervous system signaling tended to be up-regulated, specifically the synaptogenesis signaling pathway as neuron communication (Figure 4d). As IPA analysis has shown, this communication between neurons involves three regions: pre-synaptic on the tip of the axon, synaptic cleft, and post-synaptic neuron in the dendritic spine, as shown in Figure 4d. Glutamate, as a neurotransmitter can activate the intracellular signaling pathways such as LTD, LTP, and cAMP pathways in the neuron, reflected with orange shapes in this data set. Altogether, they activated the cAMP-responsive element-binding protein (CREB) as a nuclear transcription factor that triggers gene expression. In addition, some proteins marked with orange colors, such as neuronal adhesion, synaptic spine density, microtubule, and synapse stabilization, were activated in this DEG list (Figure 4d).

### 3.5. The Results of RNA-Sequencing and Gene Expression Validation

**Figure 5:**
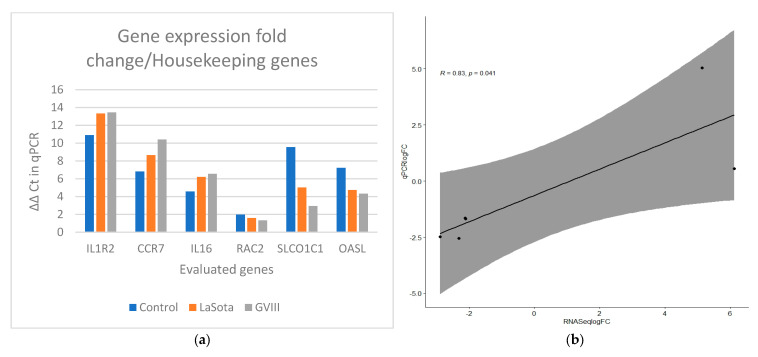
Gene expression verification results in qRT-PCRS and RNAseq. (**a**) Comparison of ΔΔCt values for vaccinated groups (orange bars for LaSota and gray bars for GVII group) with the unvaccinated group. GAPDH and ACTB were used as reference genes for normalization. (**b**) The Pearson correlation coefficient plot for the gene expression of the selected genes in vaccinated and control groups using qPCR and transcriptome analysis from RNAseq data.

Quantitative PCR was used to validate the RNA-seq results (Table 2). The correlation between qPCR results and RNA sequencing data was high (R^2^ = 0.83). Amplification efficiency (AE) of all selected genes ranges from 95% to 109% (Supplementary Table S1). In Figure 5a,b, the ΔΔCt value for each group was compared to quantify the expression using log fold change. GAPDH and ACTB were used as reference genes for normalization. The relationship between the expression of the genes from the transcriptome and qPCR was estimated using Pearson correlation.

## 4. Discussion

Using homologous or heterologous vaccines for NDV is one of the most controversial approaches in NDV control programs. Since NDV-GVII spread all around the world, vaccine failure cases have significantly increased [18]. While the NDV-GVII is the dominant strain in Southeast Asia and causing widespread outbreaks in vaccinated chickens, we have compared the immune responses of chickens that received either NDV-GVII or NDV-GII vaccines to evaluate homologous protection against NDV-GVII viruses. This study analyzed transcriptomes from the spleens of chickens vaccinated with either NDV-GII or NDV-GVII vaccines to determine the immunologic and pathophysiologic responses in chickens at the genes and pathway levels. To the best of our knowledge, this study is the first such analysis in vaccinated chickens with NDV-GVII. There were significant qualitative differences in molecular signatures between the two vaccinated groups when compared to controls. GVII-vacc modulated the canonical pathways that correlate with immune system regulation, including neuroinflammation signaling pathways and the other responses such as the synaptogenesis pathway, the effects of live NDV-GVII in infected chickens have been studied before but the effects of killed NDV-GVII vaccine a novel finding in this study [30]. It is worth mentioning that the Mega strain of NDV-GVII carries the neuropathogenic markers and causes viral encephalitis in infected chickens [29,34,49]. The spleen is always considered a key lymphatic organ in chickens, but in multiple studies, the key role of the spleen in the autonomic nervous system (ANS) that mediates, and controls the host defense has been discovered and discussed [50,51]. This significant interaction of the spleen as a lymphatic organ with the host nervous system has been highlighted in this study in many ways. Specifically, we have found, GVII-vacc inhibits the neuroinflammation signaling pathway, especially NF-ƙB as a regulator of the inflammation process but stimulates the synaptogenesis pathway to activate more intracellular signaling cells, including LTP, LTD, and cAMP in activating CREB as a transcription factor [52]. Thus, although this analysis was carried out in the spleen, it is reasonable to conclude that GVII-vacc also affects the nervous system. This finding supports the results of a previous study that this strain likely behaves as a neurotrophic virus [53]. Moreover, the connection between the peripheral nerve in the spleen with the central nucleus of the amygdala (CeA) and the paraventricular nucleus (PVN) in the brain [54] might be the bridge to trigger the signaling pathways in the brain. These conclusions haven’t been studied elsewhere and we didn’t include that in our research. It came up as one of the findings in our pathway analysis in the spleen and needs to be studied in the brain or other parts of the central nervous system in chickens. In contrast, GII did not invoke those pathways correlated with the nervous system function but instead regulated pathways that could prevent apoptosis, especially in B cells. The mechanism of apoptosis inhibition involved the down-regulation of PERK, part of EIF2α, to inhibit apoptosis-inducing ATF4/CHOP. In addition, GII-vacc inhibited cytokine signaling and leukocyte extravasation pathway as part of immune regulation in chickens. It may help to explain the reason why highly pathogenic strains of NDV like NDV-GVII triggered the HMGB1 release to promote the inflammatory response that leads to cytokine storm [55,56]. Considering all the above-mentioned pathways that we discovered in this study; it is worth discussing that there are a number of anatomical and physiological connections that exist between the immune system and the brain. The majority of interleukins have direct and indirect effects on the peripheral and central nervous system [57,58]. The Protein Network Interaction analysis in this study has confirmed that the spleen cells have significant interactions with the nervous system.

NF-ƙB was suppressed by GVII-vacc. NF-ƙB is a central component of the neuroinflammation pathway that protects the neuron system by suppressing pro-inflammation and activating neurotrophin [59,60,61]. In general, the neuroinflammation pathway plays a crucial role in maintaining homeostasis in the nervous system by strengthening synapses, sculpting circuits, and determining nervous system activity [62,63]. Usually, NF-ƙB stimulates the expression of genes encoding pro-inflammatory proteins, i.e., IL6, TNFα, IL-1β, IL18, and IL12. The down-regulation of NF-ƙB and its effects on interleukins and IFN γ, can explain the neuropathogenicity of velogenic neurotropic pathotypes of NDV [54,64,65]. In pathological conditions, increased pro-inflammatory cytokines such as IL-1β, IL6, and TNFα cause brain damage due to ischemia associated with BBB disruption [66,67,68]. However, this analysis has shown that down-regulated NF-ƙB suppresses IL-1β, IL6, and TNFα so that BBB disruption, which results in neuronal damage, is inhibited (Figure 4a). Pro-inflammatory factors IL-12 and IL-18 were inhibited in chicken vaccinated with genotype VII leading to inhibit T cell recruitment into the nervous system. Also, IFN γ in splenocytes was down-regulated (Figure 4a), reducing T cell recruitment, microglia activation, CD4 +, and CD8 + cell activation. Interestingly, the significant role of highly pathogenic NDVs in the stimulation of IFN γ has been studied by Susta et al. (2013) and showed similar pathways in interfering with the pathogenicity of NDVs in chickens [69]. Intracellular adhesion, which also plays a role in T cell recruitment in the neuronal system, is also inhibited due to down-regulated NF-ƙB in astrocytes (Figure 4a). On the other hand, the down-regulated NF-ƙB stimulates downstream neurotrophin activation, such as NTF3, indirectly activating neuron survival proteins shown with orange shape (Figure 4a). NTF3 is a neuronal protector, preventing apoptosis in cortical neurons [61]. Moreover, pathway components CASP8 and CASP3 were down-regulated to deactivate proteins that cause neuron damage and apoptosis. In a recent study Singleton et al. (2020) have shown the oncolytic effects of killed NDV vaccine on tumor cells, the most likely pathways for this effect could be through CASP8 and CASP3 and apoptosis [70]. Some studies revealed that down-regulated NF-ƙB has a role as a neuron protector by reducing inflammation and pain due to several nervous diseases, such as autoimmune encephalitis [59,60]. Thus, we speculated that the down-regulated NF-ƙB has a neuroprotective role by preventing BBB disruption from leukocytes and cytokines and increasing the production of proteins useful for neuron survival.

Another significant pathway influenced by GVII-vacc was the synaptogenesis signaling pathway. The synaptogenesis pathway controls how neurons communicate with each other, including astrocytes and microglia, using neurotransmitters [62]. In this study, the neurotransmitter was glutamate, an excitatory neurotransmitter in the nervous system [71]. Glutamate is released in the synaptic cleft and interacts with its receptors in post-synaptic neurons. The glutamate receptor has two classes, known as metabotropic glutamate receptor (mGluR) and ionotropic glutamate receptor (iGluR) [72]. iGluR class receptors such as N-Methyl-D-Aspartate Receptor (NMDAR) and α-amino-3-hydroxy-5-methyl-4-isoxazole Propionic Acid Receptor (AMPAR) at postsynaptic neurons were affected in chickens vaccinated with the GVII-vacc (Figure 4d) [73]. Such interactions in pathogenic viruses have been studied in the nervous system. In 2015, research showed the clear interaction of pathogenic influenza viruses with the genes involved in neuronal disorders. It shows even if the viruses are not directly invading the brain, they can alter the expression of the genes or signaling pathways that are directly involved in synapse assembly, neuron projection, and synaptogenesis signaling of the nervous system (CNS) [74]. NMDAR was deactivated while AMPAR was activated, possibly to maintain neuronal homeostasis, and when both receptors are activated, neurotoxicity results [75]. In addition, activation of the NMDAR has a role in excitotoxic neuronal death by increasing the Ca^2+^ influx in cells [76]. Also, excessive glutamate causes neurotoxicity [77]. In this dataset (not depicted), GRM7, a metabotropic glutamate receptor, was up-regulated and would capture the excessive amounts of glutamate at the cleft synapse and brought into pre-synaptic neurons by the solute carrier family, namely SLC1A and SLC17A. In GVII-vacc, when neurotransmitters interacted with their targets on post-synaptic neurons, these interactions activated intracellular signaling pathways, including LTD, LTP, calcium signaling, and cAMP signaling pathways. Altogether these pathways activate the transcription factor, CREB. LTP is expressed after phosphorylation of AMPAR by caMK II [78,79]. Initially, NMDAR in post-synaptic neurons removes Mg^2+^ ions to open the channel for Ca^2+^ so that calcium from the extracellular enters and activates intracellular Ca^2+^-dependent signaling [80]. Ca^2+^ contributes to post-synaptic kinase/phosphatase signaling balance that can regulate LTP and LTD [81]. CREB is a transcription factor activated by binding between Ca^2+^ and caMK II through PKA/cAMP signaling [82,83]. CREB affects long-term synaptic efficacy and is predicted to play a role in cell neuron survival [84]. However, for LTP, TMM is expressed after AMPAR dephosphorylation by protein phosphatase-1 (PP1) [85]. Cooperation between LTP and TMM with their opposite functions is crucial in synaptic plasticity [86]. We contemplated that regulation of neuron interactions and the balance of glutamate as a neurotransmitter in stimulating other intracellular signaling pathways such as CREB, LTP, and LTD pathways are the properties of the genotype VII vaccine in maintaining the nervous system homeostasis.

We predicted that the down-regulated neuroinflammation signaling pathway and the up-regulated synaptogenesis signaling pathway are hallmarks of the GVII-vacc. With down-regulated neuroinflammation, it activates proteins that play a role in neuronal survival, and by activating synaptogenesis, the balance of homeostasis in the nervous system can be maintained. Therefore, the use of the vaccine from genotype VII could be a consideration in preventing NDV outbreaks in countries where genotype VII of NDV is endemic. Furthermore, the evidence at the molecular level in this study supports evidence from previous studies in serology and clinical sign level that the genotype VII vaccine as a homologous vaccine effectively prevents an NDV outbreak [10,39,87].

In contrast, GII-vacc affected the immune response, including the cytokine signaling pathway and other responses that are not involved with the nervous system, such as cellular stress and injury genes pathway in chickens. The UPR pathway, which correlates to apoptosis, was down-regulated in GII-vacc DEG. This pathway regulates Endoplasmic Reticulum (ER) stress conducted by three transducers: PERK, IRE1, and ATF6, as a physiological or pathological response [88,89]. These three transducers participate in the apoptosis process. However, PERK as the central regulator of ER stress will determine the fate of cells due to their excessive stimulation [88]. In this GII-vacc DEG list, PERK, which is part of the EIF2α, was down-regulated, suppressing downstream ATF4/CHOP and preventing apoptosis, especially in B cells. Inhibition of apoptosis may be an advantage of the genotype II vaccine in preventing severe necrosis in lymphoid tissue and gastrointestinal, unlike the action of NDV genotype VII in a previous study [28]. Moreover, proteins encoded by genes such as XBP-1, IL-6, and cdc42, which have roles in the development, differentiation, and survival of plasma cells, were down-regulated by GII-vacc. For UPR, XBP-1 splicing mediated by IRE1 can induce differentiation of B cells, resulting in immunoglobulin expression and inducing IL-6 synthesis and secretion for plasma cell survival [90]. In our study, however, IRE1 and XBP-1 were down-regulated. Besides Rac and Rho, cdc42 is a part of the GTPases of the Rho family that regulates actin cytoskeleton dynamic, including in leukocytes [91,92]. Cdc42 is a pivotal regulator for B cell differentiation into plasma cells and the production of humoral antibodies [93]. However, in GII-vacc DEG, cdc42 was down-regulated. Deficiency of cdc42 in B cells reduces mature B cell motility and their ability to interact with T cells and inhibits B cell differentiation into antibody-producing cells [94]. Thus, the down-regulated UPR, cytoskeleton regulation, and GTPase signaling were molecular signatures of GII-vacc. Considering the significant neuroprotective activity of NDV-GVII-vacc, and the neuropathogenicity of NDV-GVII, it has been suggested that it is worth studying the transcriptomics analysis of CNS tissues in vaccinated and challenged chickens with the homologous (GVII-vacc) and heterologous vaccines [65,95].

The molecular signatures of both vaccines were predicted by using IPA software. The database of knowledge in this software is derived from the accumulated knowledge of humans and mice. When these signatures are used in other species, caution should be used in interpreting the results. Further investigations are needed to validate the role of predicted genes and pathways. For example, GVII-vacc behaves differently in terms of the effect on the nervous system in chickens, including the synaptogenesis pathway in maintaining homeostasis among neurons. However, how synaptogenesis is involved in nervous system protection if there is an NDV infection needs further study. Another prediction that arose concerned XBP-1, IL-6, and cdc42 correlated with plasma cell differentiation and survival in producing antibodies, and in chickens vaccinated using the NDV genotype II vaccine were down-regulated. This prediction raised interesting concerns regarding antibody production.

## 5. Conclusions

In conclusion, based on predictions from transcriptomic data sets using IPA software, the key difference is that the GVII-vacc activated neuroprotective activity, whereas the GII vaccine did not. Considering the NDV GVII strains are becoming dominant strains of NDV worldwide, choosing the most protective vaccines will provide better protection for chickens in the poultry industry worldwide [96]. Therefore, the application of the NDV vaccine from LaSota should not be considered in areas where GVII is the dominant genotype that causes outbreaks in the field. By revealing how both NDV vaccines interacted with the host at the gene level, this work provides an opportunity to produce new vaccines that affect specific genes or pathways essential to the chicken immune system. In addition, several genes or molecules that affect chicken immunity can be used as vaccine biomarkers to determine a vaccine’s efficacy.

## Figures and Tables

**Table 1:** The RNA-seq reads alignment to the chicken genome (GRCg6a).

Sample	Raw Reads	Cleaned Reads	Mapping (%)
Control 1	29,105,264	26,879,254	88.34
Control 2	113,657,819	102,102,063	87.89
Control 3	41,358,926	38,723,858	88.54
GII-Vacc 1	114,175,518	106,868,298	88.4
GII-Vacc 2	50,698,778	47,722,191	89.19
GII-Vacc 3	92,323,607	86,455,927	87.95
GVII-Vacc 1	158,700,330	150,616,087	89.4
GVII-Vacc 2	135,532,995	113,542,784	89.59
GVII-Vacc 3	171,523,812	157,323,249	88.76

**Table 2:** Primers for Validation of RNA-seq Results with qPCR.

NCBI Accession Number	Gene Name	Primer		Ta (°C)	AE (%)	Size (bp)	Ref.
		Forward	Reverse				
NM_205424.1	ANOS1	CCAAAGCTTCTGTGAGCCTCT	TGGGAACTTGGCATGTGTGA	60	98.5	224	This study
NM 001198752.1	CCR7	GACCATGGACGGCGGTAAAC	CGGTGACGTTGTTCCCAGCA	60	107.17	89	This study
NM_001277996.1	IL16	GCTTCAGTCTGGAAGGTGG	TGTTCCAACGAGGTCCCTTT	58	97	88	
XM_416914.6	IL1R2	AGGATGCAGAACCACAGATTTCA	CAGGTTCTCCGTGCAGTTCA	60	108.7	205	This study
XM_015293006.2	OASL	GGAGTCAGCATCACCAGTCC	CTGAATCACCTGCCCCAGTG	64	109	144	
NM_001039097.1	SLCO1C1	CCAGTGCACTCAGATACGTG	CCGAAGAACCCACAGGACAG	60	99.8	92	This study
NC_006088.5	RAC2	AGGATTACGACAGGCTGAGGC	GATGCTGGGCTGACAAGGGA	61	110	82	
AB495656.1	ACTB	CCAGACATCAGGGTGTGATGG	CTCCATATCATCCCAGTTGGTGA	60	95	137	
NC_006088.5	GAPDH	GAAGGCTGGGGCTCATCTG	CAGTTGGTGGTGCACGATG	60	104.93	150	

## Data Availability

The RNA-Seq data is available in the NCBI SRA database under the BioProject PRJNA675698.

## Supplementary Materials

The following supporting information can be downloaded at: https://www.mdpi.com/article/10.3390/pathogens13080638/s1, The RNA-Seq data is available in the NCBI SRA database under the BioProject PRJNA675698. Supplementary Table S1 contains the most up-and down-regulated genes from canonical pathways correlated with chickens’ immune response after vaccination with GII-vacc or GVII-vacc. The high-quality Figure 2, Figure 4 and Figure 5 plus the heatmap and volcano plots are available as a Supplementary File.
