# Supplementary material for: mRNA Profiling and Transcriptomics Analysis of Chickens Received Newcastle Disease Virus Genotype II and Genotype VII Vaccines
Source: Pathogens. 2024 Jul 30;13(8):638. doi: 10.3390/pathogens13080638 (PMC11357267; doi:10.3390/pathogens13080638)
Supplement: Supplementary file 1 [file pathogens-13-00638-s001.zip › Supplementary Table S1.pdf]

Supplementary Table S2. The most up-and down-regulated genes from canonical pathways correlated with chickens' immune response after vaccination with GII-vacc or GVII-vacc.

Categories	Up-Regulated Genes	Down-Regulated Genes	P-Value
**a). Significant Pathways of DEGs GII-vacc**			
Unfolded Protein Response (UPR)	N/A	CEBPB, HSPA (5/8), HSPH (1/2)	1. 78E-06
CXCR4 signaling	N/A	CXCR4, EGR1, FOS, MYL (4/9)	3. 48E-04
IL-6 signaling	N/A	CEBPB, FOS, HSPB (1/7)	1. 09E-03
Leukocyte extravasation	N/A	ACTA1, ACTG2, CXCR4, THY1	5. 65E-03
**b). Significant Pathways of DEGs GVII-vacc**			
Neuroinflammation signaling pathway	ACVR2B, AKT3, BDNF, CREB5, CALB1, GABRA (1/2/3/4/6), GABRB (1/2/3), GABBR2, GABRG(1/2), GRIA1, GRIN (1/2A/2B), KCNJ6, NTF3, NOX3, PIK3C2G, PLA2 (G4B/G4E/G4F/G10), SNCA, SLC (1A3/6A1/6A11), TGFB2	CCL5, CYBB, CX3CR1, CRP, DRA, FOS, HLA-A, HLA-IL1R1, JAK1, P2RX7, PIK3R5, TREM2, TLR (2/7)	1.11E-07
Leukocyte extravasation signaling	ACTN2, AFDN, BMX, CDH5, CLDN (1/10), CTNNA (2/)3, DLC1, EDIL3, MMP16, NOX3, PIK3C2G, PRKCE, TIMP4	ACTA1, ACTG2, CYBB, ITGB3, MMP9, PIK3R5, PRKCH, PTK2B, RHOH, RAC2, THY1	1.57E-07
IL-15 production	BMX, EGFR, EPH (A3/A5/B2), ERBB4, FLT1, FRK, MET, MUSK, RET, ROR2, ROS1, STYK1, YES1	BTK, CSK, MATK, PTK2B, ZAP70	3.63E-06
PKCθ signaling in T lymphocyte	CACNA (1B/1C/1D/1G/1I/2D1/2D 2/2D3), CACN (G2/B2), MAP3K15, PIK3C2G	CD (3D/3E/4/28/247), CACNB4, FOS, GRAP2, HLA(A/DRA), LCP2, NFKBIE, PIK3R5, RAC2, ZAP70	4.35E-06
iCOS-iCOSL signaling in T helper cells	AKT3, CAMK4, GAB2, PIK3C2G	CD (3D/3E/4/28/40L/247), CSK, GRAP2, HLA(A/DRA), INPP5D, IL2RG, LCP2, NFKBIE, PLEKHA2, PTPRC, PIK3R5, TRAT1, ZAP70	1.20E-05
IL-9 signaling	PIK3C2G, SOCS2	IL2RG, JAK1, PIK3R5	2.65E-04
Role of NFAT in the regulation of the immune response	AKT3, CAMK4, GNAS, PIK3C2G, PLCB (1/4)	BTK, CD(3D/3E/4/28/79B/247), FOS, HLA (A/DRA), LCP2, NFKBIE, PIK3R5, ZAP70	7.49E-04

Synaptogenesis signaling pathway	ADCY (1/2/8), AKT3, BDNF, CACNA1B, CAMK4, CNTNAP2, CDH (4/6/7/8/9/10/12/13/18/19/20), CPLX1, CREB5, EPHA (3/5/7), EFNA5, GRIA (1/2/4), GRM (1/5/7/8), GRIN1, GRIN2 (A/B), NLGN (1/4Y), Nrxn3, NRXN1, PIK3C2G, PRKAR2B, SYT (1/4/9/14/17), SNCA, STXBP6, THBS2, YES1	ADCY7, ARPC1B, CACNB4, CDH3, SYT (2/8)	1.23E-20
