# Supplementary figures and images for: mRNA Profiling and Transcriptomics Analysis of Chickens Received Newcastle Disease Virus Genotype II and Genotype VII Vaccines
Source: Pathogens. 2024 Jul 30;13(8):638. doi: 10.3390/pathogens13080638 (PMC11357267; doi:10.3390/pathogens13080638)

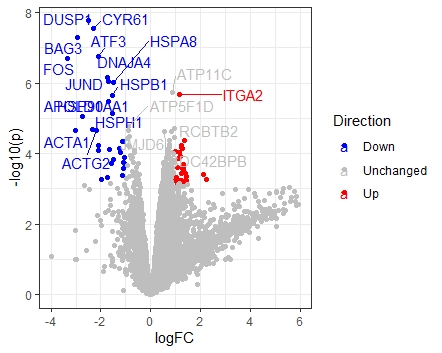

Supplement: Supplementary file 1 [file pathogens-13-00638-s001.zip › Sup Volcano Plot_20 genes GII vs NC.jpeg]

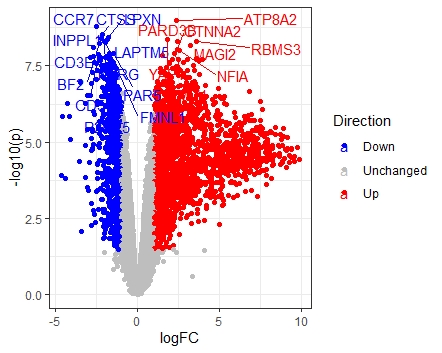

Supplement: Supplementary file 1 [file pathogens-13-00638-s001.zip › Sup Volcano Plot_20 genes GVII vs NC.jpeg]
